# Three-dimensional fully coupled hydro-mechanical-chemical model for solute transport under mechanical and osmotic loading conditions

**DOI:** 10.1007/s11356-022-22600-0

**Published:** 2022-08-20

**Authors:** Shakil A. Masum, Zhihong Zhang, Gailei Tian, Mimnun Sultana

**Affiliations:** 1 Geoenvironmental Research Centre, Cardiff University, Cardiff, CF24 3AA UK; 2 Key Laboratory of Urban Security & Disaster Engineering, Ministry of Education, Beijing University of Technology, Beijing, 100124 People’s Republic of China; 3 United International University, Dhaka, 1212 Bangladesh

**Keywords:** Osmosis, Consolidation, Coupled modelling, Compacted clay, Geomembrane, Rebound

## Abstract

Mechanical deformation and chemico-osmotic consolidation of clay liners can change its intrinsic transport properties in all direction and can alter fluid and solute transport processes in the entire model domain. These phenomena are described inadequately by lower-dimensional models. Based on the Biot’s consolidation theory, fluid and solute mass conservation equations, a three-dimensional (3D) fully-coupled hydro-mechanical-chemical (HMC) model has been proposed in this study. The impacts of mechanical consolidation and chemico-osmotic consolidation on permeability, hydrodynamic dispersion, solute sorption, membrane efficiency, and chemical osmosis are considered in the model. The model is applied to evaluate performances of a single compacted clay liner (CCL) and a damaged geomembrane-compacted clay composite liner (GMB/CCL) to contain a generic landfill contaminant. Effect of model dimensionality on solute spread for CCL is found to be marginal, but for GMB/CCL the effect is significantly large. After 50-year simulation period, solute concentration at the half-length of the GMB/CCL liner is predicted to be 40% of the source concentration during 1D simulation, which is only 6% during the 3D simulation. The results revealed approximately 74% over-estimation of liner settlement in 1D simulation than that of the 3D for GMB/CL system. Solute spread accelerates (over-estimates) vertically than horizontally since overburden load and consequent mechanical loading-induced solute convection occurs in the same direction. However, in homogeneous and isotropic soils, horizontal spread retards the overall migration of contaminants, and it highlights the importance of 3D models to study solute transports under mechanical and chemico-osmotic loading conditions in semi-permeable clays, especially, for damaged geomembrane-clay liners. The results show the utility of geomembranes to reduce soil settlement, undulation, and restriction of solute migration. Furthermore, application of geomembrane can inhibit development of elevated negative excess pore water pressure at deeper portion of a clay liner.

## Introduction

Clays and clayey soils are often used as buffer materials, such as landfill liners to protect the surrounding geology and groundwater around landfill sites. Detailed understanding of physical, chemical, and mechanical behavior of clay liners are crucial to ensure their effective and adequate performance (Chen 2014; Reddy et al. 2017). Numerous research efforts and investigations are available in literatures focusing on the material’s behavior under various physical, chemical, and mechanical loading conditions (Xie et al. 2022; Yan et al. 2021a, b, c, 2022). However, the current work is focused on understanding the osmotic behavior of clays and its impact on solute transport and settlement behaviors of landfill clay liners. This is of significant importance where landfill leachate is in direct contact with the landfill liners (Zhang et al. 2018; 2021).

Studies of contaminant (or solute) transport in clay liners, usually assume that the liners are rigid porous media, and flow of solutes are driven by advection and/or diffusion mechanisms and influenced by sorption processes (Li et al. 2002; Islam and Singhal 2002; Bourg et al. 2003; Kooi et al. 2003; Dominijanni and Manassero 2005; Zhang et al. 2005; Reddy et al. 2017; Qiu et al. 2022). However, clay liners undergo mechanical consolidation due to the weight of landfill wastes altering its intrinsic transport properties (Huang et al. 2012; Zhang et al. 2018; Zheng et al. 2021). Influences of liner consolidation on solute transport have been extensively studied in Smith, (2000); Alshawabkeh and Rahbar (2006); Zhang et al., (2013; Xie et al., (2016); Yu et al., (2018); Pu et al., (2020); Yan et al., (2021a, b, c, 2022); Qiu et al., (2022). Meanwhile, clayey soils that possess semi-permeable membrane properties promote osmosis, a process where movement of a solute occurs from higher to lower concentration region, while the solvent moves in the opposite direction in the presence of the solute concentration gradient across the semi-permeable membranes. The movement of pore fluid drives osmotic-consolidation which has been of particular interest over the past few decades. Initial theoretical developments of Greenberg et al. (1973), Barbour and Fredlund (1989), and Mitchell (1993) on chemical osmosis and osmotic-consolidation are extended and advanced in the works of Kaczmarek and Hueckel (1998), Smith (2000), Peters and Smith (2002, 2004), Malusis and Shackelford (2002), Lewis et al. (2009), Huang et al. (2012), Musso et al. (2013), Pu et al. (2020), Xie et al. (2016) and others. Zhang et al. (2018) conducted a comprehensive analysis of those models and revealed that majority of those models did not consider multiphase flow and deformation and their simultaneous interactions on osmotic processes. In other words, the models are mostly un-coupled or partially coupled in nature, which is a significant limitation, since clay liners in landfills are exposed to multiphase flows of fluids, chemicals, heat as well as mechanical deformation conditions. They are also largely limited to one-dimensional or two-dimensional problems and do not represent realistic or in situ design layouts of landfill liners. For example, mechanical deformation of clay liners can change its intrinsic transport properties in all direction, which eventually can alter fluid and solute transport processes in the entire model domain. These phenomena are described inadequately by lower-dimensional models. Within the scope of their work, Zhang et al. (2018) developed a fully coupled HMC model by addressing the limitations of the previous models, e.g., Kaczmarek and Hueckel (1998), Peters and Smith (2002, 2004), Malusis and Shackelford (2002), Musso et al. (2013), Pu et al. (2020). Nevertheless, the model of Zhang et al. (2018) was also one-dimensional. Huang et al. (2012) and Zhang and Fang (2016), Zhang et al. (2017) developed 3D coupled models describing solute transport in deforming soil taking account of the effect of consolidation on solute transport processes. However, to the authors’ knowledge, a fully coupled, three-dimensional HMC model that investigates both mechanical consolidation and osmotic-consolidation is rarely available. Accurate assessment of clay liner performance is crucial to design landfill barriers for optimum containment of chemical solutes (Chen 2014). Variation of spatial dimension can influence the migration law of solutes (Li et al. 2011). Therefore, it is essential to establish a three-dimensional model to investigate solute transport processes under complex engineering and geoenvironmental conditions.

In this work, the previously published model of Zhang et al. (2018) is further extended to develop a fully coupled 3D HMC model with the aim to overcome the above limitations and accurately represent landfill clay liners. Both mechanical consolidation, associated with landfill waste, and chemico-osmotic consolidation, driven by solute concentration gradient, are included in the model formulations. Impacts of dynamic hydraulic permeability, hydrodynamic dispersion, solute sorption, membrane efficiency, and chemical osmosis on solute transport behavior are considered in the proposed model. The finite element software COMSOL Multiphysics is used to develop the 3D numerical model. The model is applied to evaluate performances of a single compacted clay liner and a geomembrane-compacted clay liner (GMB/CCL) to contain a generic landfill contaminant (or solute).

## Coupling model and governing equation

In this section, the governing equations of the fully coupled 3D HMC model is presented. The following assumptions are made: soil is homogeneous, isotropic, and under isothermal condition; soil particles are incompressible and deformation is linear and elastic.

### Governing equation of soil deformation

Given that soil deformation is marginal, the stress equilibrium equations follow:

1 $$\begin{array}{c}\frac{\partial {\sigma }_{xx}}{\partial x}+\frac{\partial {\sigma }_{xy}}{\partial y}+\frac{\partial {\sigma }_{zx}}{\partial z}=0\\ \frac{\partial {\sigma }_{xy}}{\partial x}+\frac{\partial {\sigma }_{yy}}{\partial y}+\frac{\partial {\sigma }_{yz}}{\partial z}=0\\ \frac{\partial {\sigma }_{zx}}{\partial x}+\frac{\partial {\sigma }_{zy}}{\partial y}+\frac{\partial {\sigma }_{zz}}{\partial z}=-\gamma \end{array}$$

where ${\sigma }_{ij}$ is stress (*i*, *j* = *x*, *y*, *z*); $\gamma$ is unit weight of pore water. According to the principle of effective stress (Terzaghi 1943), the stress equilibrium can be written as:

2 $$\begin{array}{c}\frac{\partial {\sigma }_{xx}^{^{\prime}}}{\partial x}+\frac{\partial {\sigma }_{xy}}{\partial y}+\frac{\partial {\sigma }_{zx}}{\partial z}+\frac{\partial u}{\partial x}=0\\ \frac{\partial {\sigma }_{xy}}{\partial x}+\frac{\partial {\sigma }_{yy}^{^{\prime}}}{\partial y}+\frac{\partial {\sigma }_{yz}}{\partial z}+\frac{\partial u}{\partial y}=0\\ \frac{\partial {\sigma }_{zx}}{\partial x}+\frac{\partial {\sigma }_{zy}}{\partial y}+\frac{\partial {\sigma }_{zz}^{^{\prime}}}{\partial z}+\frac{\partial u}{\partial z}=-\gamma \end{array}$$

where ${\sigma }_{ij}^{^{\prime}}$ is the effective stress. For isotropic linear poroelastic medium, the constitutive relations can be written in terms of effective stress *σ*_*ij*_′, strain *ε*_*ij*_, and chemical concentration change as (Li et al. 2011; Zhang and Fang 2016) follows:

3 $$\begin{array}{c}{\sigma }_{xx}^{^{\prime}}=2G\left(\frac{\nu }{1-2\nu }{\varepsilon }_{v}+{\varepsilon }_{xx}\right)-{\alpha }_{c}\frac{E}{1-2\nu }\left(c-{c}_{s}\right)\\ {\sigma }_{yy}^{^{\prime}}=2G\left(\frac{\nu }{1-2\nu }{\varepsilon }_{v}+{\varepsilon }_{yy}\right)-{\alpha }_{c}\frac{E}{1-2\nu }\left(c-{c}_{s}\right)\\ \begin{array}{c}{\sigma }_{zz}^{^{\prime}}=2G\left(\frac{\nu }{1-2\nu }{\varepsilon }_{v}+{\varepsilon }_{zz}\right)-{\alpha }_{c}\frac{E}{1-2\nu }\left(c-{c}_{s}\right)\\ {\sigma }_{yx}=G{\varepsilon }_{yx},{\sigma }_{zx}=G{\varepsilon }_{zx},{\sigma }_{yz}=G{\varepsilon }_{yz}\end{array}\end{array}$$

where *α*_c_ = *m*_*c*_(1 − $\nu$)/( 1 + $\nu$), and *m*_*c*_ is the coefficient of volume change due to chemical concentration change; *G* is shear modulus of soil medium; $\nu$ is Poisson’s ratio; *E* is elastic modulus of soil medium; ${c}_{s}$ is the concentration of contaminant in the external environment; $c$ is the concentration of contaminant in soil; ${\varepsilon }_{v}$ is volume strain; ${\varepsilon }_{ij}$ is the strain. In addition, the strain–displacement relations can be written as follows:

4 $$\begin{array}{c}{\varepsilon }_{xx}=-\frac{\partial {w}_{x}}{\partial x},{\varepsilon }_{yz}=-\left(\frac{\partial {w}_{y}}{\partial z}+\frac{\partial {w}_{z}}{\partial y}\right)\\ {\varepsilon }_{yy}=-\frac{\partial {w}_{x}}{\partial x},{\varepsilon }_{zx}=-\left(\frac{\partial {w}_{z}}{\partial x}+\frac{\partial {w}_{x}}{\partial z}\right)\\ {\varepsilon }_{zz}=-\frac{\partial {w}_{x}}{\partial x},{\varepsilon }_{xy}=-\left(\frac{\partial {w}_{y}}{\partial x}+\frac{\partial {w}_{x}}{\partial y}\right)\end{array}$$

where *w*_*i*_ is the displacement in the *i*-direction (*i* = *x*, *y*, *z*).

Substituting Eqs. (2)–(4) yields the governing equation of soil deformation:

5 $$\begin{array}{c}-G{\nabla }^{2}{w}_{x}-\frac{G}{1-2\nu }\frac{\partial }{\partial x}\left(\frac{\partial {w}_{x}}{\partial x}+\frac{\partial {w}_{y}}{\partial y}+\frac{\partial {w}_{z}}{\partial z}\right)-{\alpha }_{c}\frac{E}{1-2\nu }\frac{\partial c}{\partial x}+\frac{\partial u}{\partial x}=0\\ -G{\nabla }^{2}{w}_{y}-\frac{G}{1-2\nu }\frac{\partial }{\partial y}\left(\frac{\partial {w}_{x}}{\partial x}+\frac{\partial {w}_{y}}{\partial y}+\frac{\partial {w}_{z}}{\partial z}\right)-{\alpha }_{c}\frac{E}{1-2\nu }\frac{\partial c}{\partial y}+\frac{\partial u}{\partial y}=0\\ -G{\nabla }^{2}{w}_{z}-\frac{G}{1-2\nu }\frac{\partial }{\partial z}\left(\frac{\partial {w}_{x}}{\partial x}+\frac{\partial {w}_{y}}{\partial y}+\frac{\partial {w}_{z}}{\partial z}\right)-{\alpha }_{c}\frac{E}{1-2\nu }\frac{\partial c}{\partial z}+\frac{\partial u}{\partial z}=0\end{array}$$

where $\nabla$ is the Laplacian,$\nabla =\frac{{\partial }^{2}}{\partial {x}^{2}}+\frac{{\partial }^{2}}{\partial {y}^{2}}+\frac{{\partial }^{2}}{\partial {z}^{2}}$.

### Governing equation of pore fluid flow

The flow continuity equation can be expressed as follows:

6 $$\frac{\partial \left({\rho }_{f}n\right)}{\partial t}+\nabla \cdot \left({\rho }_{f}n{{{q}}}_{{{f}}}\right)=0$$

where *ρ*_*f*_ is the density of the pore fluid; *n* is porosity and ***q***_*f*_ is the absolute velocity of the pore fluid. By considering that the pore fluid is incompressible, Eq.(6) yields:

7 $$\frac{\partial n}{\partial t}+\nabla \cdot \left(n{{{q}}}_{{{f}}}\right)=0$$

Osmotic potential driven by solute concentration gradient across semi-permeable clay liner may lead to soil deformation and change in porosity. Following Kaczmarek and Hueckel (1998), Musso et al. (2013), and Zhang et al. (2018), the change of porosity can be expressed as follows:

8 $$n={n}_{0}-\Delta n={n}_{0}+{m}_{v}u-{m}_{\pi }c$$

where *m*_*v*_ is the coefficient of volume change due to the effective stress, ${m}_{\pi }$ and is the coefficient of volume change due to osmotic potential. Substituting Eq. (8) into Eq. (7) yields:

9 $${m}_{v}\frac{\partial u}{\partial t}-{m}_{\pi }\frac{\partial c}{\partial t}+\nabla \cdot \left(n{{{q}}}_{{{f}}}\right)=0$$

The absolute velocity of the pore fluid ***q***_*f*_ can be written as follows:

10 $${{{q}}}_{{{f}}}-{{{v}}}_{{{s}}}={{{q}}}_{{{r}}}$$

where ${{{v}}}_{{{s}}}$ is the velocity of soil skeleton and can be written as follows:

11 $${{{v}}}_{{{s}}}=\frac{\partial {{w}}}{\partial t}$$

where ***w*** is the displacement tensor and can be written as ***w*** = (*w*_*x*_, *w*_*y*_, *w*_*z*_)^T^.

***q***_*r*_ is the pore fluid velocity relative to the soil skeleton and it can be expressed as follows:

12 $${{{q}}}_{{{r}}}=\frac{1}{n}{{q}}$$

where ***q*** is the Darcy velocity of the pore fluid and can be written as ***q*** = (*q*_*x*_, *q*_*y*_, *q*_*z*_)^T^. Zhang et al. (2018) considered the influence mechanical loading and osmotic potential on ***q*** as follows:

13 $$\begin{array}{c}{q}_{x}=\left(1-\omega \right)\left(-\frac{{k}_{x}}{{\gamma }_{w}}\frac{\partial u}{\partial x}+\omega \frac{{k}_{x}}{{\gamma }_{w}}\frac{RT}{M}\frac{\partial c}{\partial x}\right)\\ {q}_{y}=\left(1-\omega \right)\left(-\frac{{k}_{y}}{{\gamma }_{w}}\frac{\partial u}{\partial y}+\omega \frac{{k}_{y}}{{\gamma }_{w}}\frac{RT}{M}\frac{\partial c}{\partial y}\right)\\ {q}_{z}=\left(1-\omega \right)\left(-\frac{{k}_{z}}{{\gamma }_{w}}\frac{\partial u}{\partial z}+\omega \frac{{k}_{z}}{{\gamma }_{w}}\frac{RT}{M}\frac{\partial c}{\partial z}\right)\end{array}$$

where *ω* is osmotic efficiency; *R* is universal gas constant; *T* is the absolute temperature, *k*_*x*_, *k*_*y*_, and *k*_*z*_ are the permeability coefficients in *x*, *y*, and *z* directions. When the permeability of soil is isotropic, average permeability *k* = *k*_*x*_ = *k*_*y*_ = *k*_*z*_. Hart and John (1986) expressed in situ hydraulic conductivity in relation to the initial hydraulic conductivity and initial porosity as follows:

14 $$k={k}_{0}{\left(\frac{n}{{n}_{0}}\right)}^{3}$$

where *k*_0_ is the initial hydraulic conductivity and *n*_0_ is initial porosity.

Substituting Eqs. (11) to (13) into Eq. (10) results:

15 $$n{{{q}}}_{{{f}}}={{q}}+n\frac{\partial {{w}}}{\partial t}$$

Finally, substituting Eq. (15) into Eq. (9), the governing equation of pore fluid flow yields:

16 $${m}_{v}\frac{\partial u}{\partial t}-{m}_{\pi }\frac{\partial c}{\partial t}+\nabla \cdot \left({{q}}+n\frac{\partial {{w}}}{\partial t}\right)=0$$

### Governing equation of solute transport

Following the principle of mass conservation, solute transport in pore fluid can be written as follows:

17 $$\frac{\partial\left(nc\right)}{\partial t}=-\nabla\cdot J_f\pm Y$$

where *Y* is the sink/source; *J*_*f*_ represents the solute flux tensor in the pore fluid. The term ***J***_***f***_ can be expressed as follows:

18 $${{{J}}}_{{{f}}}={{{J}}}_{{{a}}}+{{{J}}}_{{{D}}}={{{J}}}_{{{a}}}+{{{J}}}_{{{m}}}+{{{J}}}_{{{d}}}$$

in which *J*_*a*_ is the solute convective flux tensor; *J*_*D*_ is the solute hydrodynamic dispersion flux tensor; *J*_*m*_ is the solute chemical diffusion flux tensor; *J*_*d*_ is the solute mechanical dispersion flux tensor.

The solute convective flux tensor is given by the following:

19 $${{{J}}}_{{{a}}}=n{{q}}c$$

The solute chemical diffusion flux tensor can be expressed as follows:

20 $${{{J}}}_{{{m}}}=-n{D}_{m}\nabla c$$

where *D*_*m*_ represents the effective diffusion coefficient tensor of contaminant in porous medium and can be defined as (Malusis et al. 2014) follows:

21 $${D}_{m}=\left(1-\omega \right)\tau {D}_{0}$$

where *D*_0_ is the diffusion coefficient for the contaminant in a free solution; ***τ*** is the tortuosity factor for soil and can be determined by the empirical formula, ***τ*** = (*τ*_*x*_, *τ*_*y*_, *τ*_*z*_)^T^ = (*n*^*m*^, *n*^*m*^, *n*^*m*^)^T^; *m* is the empirical parameter (Liu et al. 2004).

The solute mechanical dispersion flux can be written as follows:

22 $${{{J}}}_{{{d}}}=-n{D}_{d}\nabla c$$

where *D*_*d*_ is the mechanical dispersion coefficient tensor and can be expressed as follows:

23 $${D}_{d}={\alpha }_{L}\left|q\right|$$

where ***α***_*L*_ is the dispersivity tensor and can be written as ***α***_*L*_ = (*α*_*T*_, *α*_*T*_, *α*_*L*_)^T^, *α*_T_ is transverse dispersion, and *α*_*L*_ is longitudinal dispersion. In practice, the combination of molecular diffusion and mechanical dispersion is called hydrodynamic dispersion, and the hydrodynamic dispersion coefficient tensor can be written as follows:

24 $$D={D}_{d}+{D}_{m}$$

where *D* is the hydrodynamic dispersion coefficient tensor. The solute hydrodynamic dispersion flux tensor can be expressed as follows:

25 $${{{J}}}_{{{D}}}=-nD\nabla c$$

Substituting Eqs. (18), (19), and (25) into Eq. (17) yields:

26 $$\frac{\partial \left(nc\right)}{\partial t}=\nabla \cdot \left(nD\cdot \nabla c-n{{q}}c\right)\pm Y$$

Similar to solute transport in pore fluid, the mass conservation equation of a solute in the solid phase can be written as follows:

27 $$\frac{\partial\left[\left(1-n\right)\rho_sS\right]}{\partial t}=-\nabla\cdot J_s\pm Y$$

where ***J***_*s*_ is the solute flux tensor in soil particles and *S* is the mass of contaminant adsorbed within the soil particles per unit mass of solid. Considering the adsorption isotherm is liner:

For convenient calculation, the linear isothermal adsorption has been occupied as follows:

28 $$S={K}_{d}c$$

The flux ***J***_*s*_ is described by the following:

29 $${{{J}}}_{{{s}}}=\left(1-n\right){{{v}}}_{{{s}}}{\rho }_{s}S$$

Substituting Eqs. (28)–(29) into Eq. (27) yields:

30 $$\frac{\partial \left[{K}_{d}\left(1-n\right){\rho }_{s}c\right]}{\partial t}=-\nabla \cdot \left[{K}_{d}\left(1-n\right){\rho }_{s}c\frac{\partial {{w}}}{\partial t}\right]\pm Y$$

Assume that the sink/source in the sorption process is the same as that in the desorption process. Equations (26) and (30) can be combined to achieve the governing equation of solute transport as follows:

31 $$\frac{\partial \left(nc\right)}{\partial t}+\frac{\partial \left[{K}_{d}\left(1-n\right){\rho }_{s}c\right]}{\partial t}=\nabla \cdot \left(nD\cdot \nabla c-n{{q}}c\right)-\nabla \cdot \left[{K}_{d}\left(1-n\right)\frac{\partial {{w}}}{\partial t}{\rho }_{s}c\right]$$

Equations (5), (16), and (31) constitute the three-dimensional, fully coupled HMC model equations for solute transport including osmotic and mechanical loading in saturated, semi-permeable clays. The main variables in the fully coupled equation include the displacement vector ***w***, the excess pore water pressure *u*, and the chemical concentration *c*. In addition, the use of the coupling parameters *n*, ***q****,* and *D* affected by mechanical load and solute concentration enables the governing equation to realize the full coupling process.

## Model verification/evaluation

Laboratory experimental data to validate the 3D coupled model is not currently available in literatures. Therefore, in this section, the proposed model is evaluated against the analytical solution of Yan et al. (2021a). The model is built with the COMSOL Multiphysics (https://www.comsol.com) software. Yan et al. (2021a) derived analytical solutions for transient contaminant transport in clay liner under the combined effects of diffusion, adsorption, and consolidation processes. However, the semipermeable membrane property, chemical osmotic flow, and chemico-osmotic consolidation were ignored. After the necessary simplification, numerical calculation was carried out, and the predicted results of solute concentration across the depth of the liner were compared the analytical solutions of Yan et al. (2021a). The initial and boundary conditions and the model parameters are listed in Tables 1 and 2, respectively. The discretized model domain contains 0.114-m linear segments and time-steps of 36.5 days.

**Table 1:** Initial and boundary conditions of the validation test

Initial conditions	$c\left(z,0\right)=0$	$e\left(z,0\right)=$ 1.2	$\left(0<z<2m\right)$
Boundary conditions	$c\left(0,t\right)={c}_{0}$	$\frac{\partial e}{\partial x}\left(0,t\right)=$ 0	$\left(t\ge 0\right)$
	$\frac{\partial c}{\partial x}\left(L,t\right)=0$	$e\left(L, t\right)={e}_{p}-{C}_{c}\mathrm{log}(\frac{{\sigma }_{a}}{{\sigma }_{p}^{\mathrm{^{\prime}}}})$	$\left(t\ge 0\right)$

**Table 2:** Simulation parameters of the validation test. Data collected from Yan et al. (2021a)

Parameters	Values
Contaminant concentration, *c*_0_	1 g·m^−3^
Diffusion coefficient in the CCL, *D*	0.03 m^2^·a^−1^
Clay sorption, ${{\rho}}$ _*d*_*Kd*	0
Initial void ratio, *e*_0_	1.2
Coefficient of compressibility, *a*_*v*_	6.95 MPa^−1^
Unit weight of water, *γ*_*w*_	10 kN·m^−2^
Compression index, *C*_*c*_	0.8
Hydraulic conductivity, *k*_0_	0.01 m·a^−1^
Maximum applied stress, ${{{\sigma}}}_{{{a}}}$	500 kPa
Pre-consolidation stress, ${{{\sigma}}}_{{{p}}}^{\mathbf{^{\prime}}}$	50 kPa

**Fig. 1:**
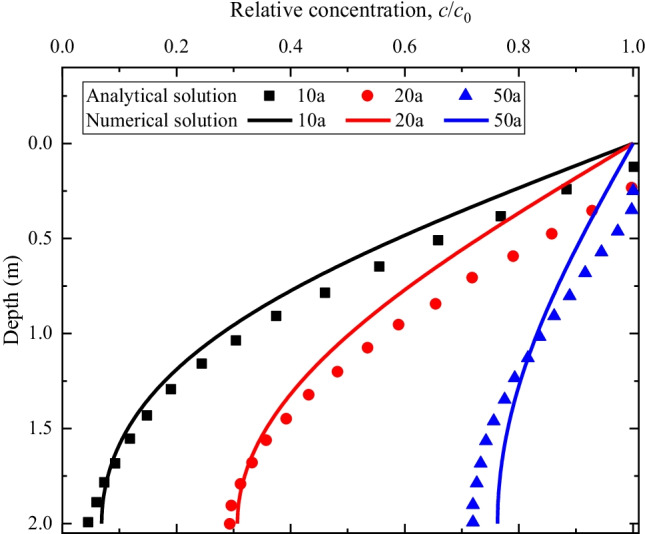
Comparison of the model predicted solute concentration results with that of the analytical solution of Yan et al. (2021a)

The simulation results and their comparison with the analytical solutions of Yan et al. (2021a) are presented in Fig. 1. It is observed that the model predicted results are in good agreement with the analytical solution of Yan et al. (2021a), which demonstrates accuracy and reliability of the proposed model to study contaminant transport in clay liner involving simultaneous reactive transport and mechanical deformation processes.

## Model application

**Fig. 2:**
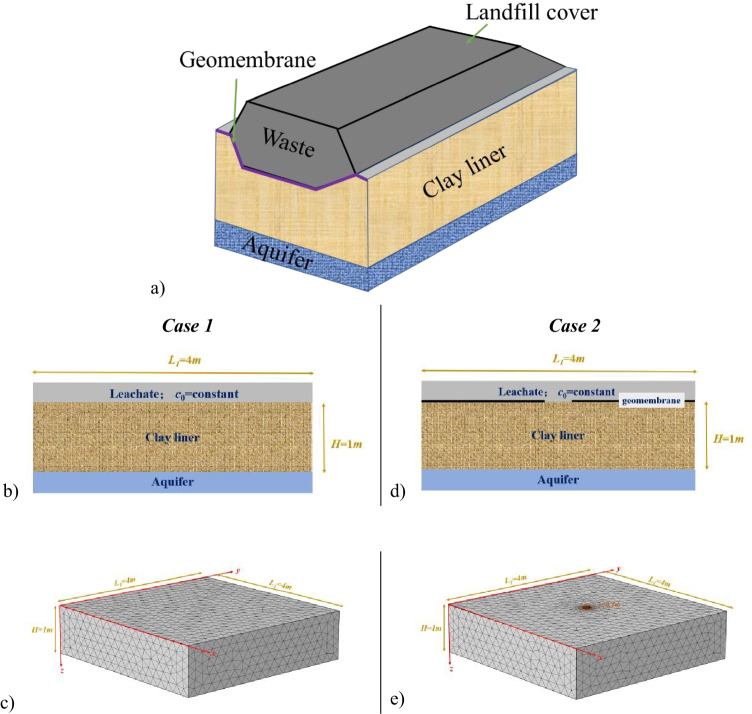
(**a**) Representative sketch of a typical 3D landfill model. (**b**) Idealized model and (**c**) discretized 3D model domain of compacted clay liner or CCL. (**d**) Idealized model and (**e**) discretized 3D model domain of geomembrane-compacted clay composite liner GMB/CCL

Landfill liner system often used to restrict/contain leachate and solutes from seeping into the surrounding geology or groundwater. Single compacted clay liner and GMB/CCL are always used as the barrier system, and their service life are generally longer than the operation period of landfill site and the stabilization time of solid waste (which could be up to 50 years). In this section, the proposed model is used to predict evolution of excess pore water pressure, soil deformation, and solute migration behavior under the combined action of mechanical loading, and chemical loading in single compacted clay liner (CCL) and geomembrane-compacted clay liner (GMB/CCL) systems. When compacted clay is used as the contaminant barrier, CCL is directly exposed to leachate. Although GMB/CCLs provide higher containment than CCL, they often experience unwanted damage during installation in the landfill sites. By analyzing field data, Rowe and Brachman (2004) concluded that 70% of the sites reported torn or ripped geomembranes, even though the construction quality was strictly controlled (Bouazza, 2002; Rowe and Brachman 2004; Brachman and Gudina 2008). Therefore, in this study, two possible scenarios are investigated where a clay liner is exposed directly to the leachate (Case 1) or through a damaged portion of a GMB/CCL (Case 2). A conceptual physical system is illustrated in Fig. 2a, and the idealized model is presented in Fig. 2b and d for CCL (Case 1) and damaged or leaked GMB/CCL (Case 2), respectively.

The model domain is 4 m × 4 m × 1 m (Ω _1_) which contains a circular leakage (Ω _2_) of 0.1 m radius at the center of the top surface in Case 2 (Fig. 2d). The model parameters are listed in Table 3. In this study, a low stress or applied waste load of 50 kPa is considered following Woodman et al. (2014) and Das and Bharat (2021) as well as to be consistent with the 1D simulations presented in Zhang et al. (2018) for allowing comparisons between 1 and 3D model predictions. Following Kaczmarek and Hueckel (1998), in this study, it is assumed that the application of mechanical load is instantaneous meaning soil has no time to deform and the applied load is accommodated by the pore fluid. This is reflected at the initial pore fluid pressure considered in the simulations. The 3D model domain is discretized using tetrahedra mesh elements. For the Case 1 simulation, Fig. 2c, uniform mesh elements of 0.14 m is used. Whereas, in Fig. 2e, near the circular leakage, mesh element varies between 0.0008 and 0.08 m and in the rest of the domain between 0.0006 and 0.14 m. The simulation runtime is 50 years, and the discretized time-step between 0 and 1 year is 3.65 days and, between 1 and 50 years, it is 36.5 days.

**Table 3:** Model parameters

Parameters	Value	Reference
Initial permeability coefficient, *k*_0_	1 × 10^−10^ m·s^−1^	Kaczmarek and Hueckel (1998)
Coefficient of volume, *m*_*v*_	5 × 10^−7^ m·s^2^·kg^−1^	,,
Coefficient of volume, *m*_*c*_	0.105 × 10^−3^m^3^·kg^−1^	,,
Initial porosity, *n*_0_	0.5	Peters and Smith (2004)
The density of the dry soil, *ρ*_*s*_	2.6 × 10^3^ kg·m^−3^	,,
The density of the pore fluid, *ρ*_*w*_	1 × 10^3^ kg·m^−3^	,,
Effective diffusion coefficient, *D*_*m*_	5.01 × 10^−10^ m^2^·s^−1^	,,
Poisson ratio, *v*	0.3	Zhao and Bai (2004)
Shear modulus, *G*	2.6 × 10^3^ kPa	Bengtsson and Sällfors (1983)
Longitudinal dispersion, *α*_L_	0.001 m	Zhang et al. (2015)
Transverse dispersion, *α*_T_	0.01 m	,,
Empirical constant, *m*	2.96	Fitted
Osmotic efficiency, *ω*	0.03	Given
Universal gas constant, *R*	8.314 J·mol^−1^·K^−1^	
Molar mass of NaCl, *M*	0.0585 kg·mol^−1^	
Linear adsorption coefficient, *k*_*d*_	0.8142 × 10^−3^ m^3^·kg^−1^	Zhang et al. (2018)

The clay liner properties, of the presented simulations, are obtained from Kaczmarek and Hueckel (1998) and Peters and Smith (2004). The model application example of Kaczmarek and Hueckel was extended by Peters and Smith to study NaCl leachate through a typical landfill liner. However, additional parameters were required to support the model simulations presented in this study. For example, the shear modulus data was obtained from Bengtsson and Sällfors (1983), who measured the value for soft, plastic Swedish clays representative of a typical landfill clay liner. The longitudinal and transverse dispersion coefficients, for a natural clay liner, were obtained from Zhang et al. (2015), and the empirical parameter, *m*, to calculate tortuosity (Eq.21), was obtained from the effective diffusion coefficient and porosity data.

## Results and discussion

### Barrier system including single compacted clay liner (Case 1)

In this case, the CCL is directly exposed to leachate, and the solute concentration at any point on the upper boundary (*z* = 0 m) of the liner is same as that in the leachate. The initial and boundary conditions of the simulations are presented in Table 4.

**Table 4:** Initial and boundary conditions of the Case 1 (CCL) simulation scenario

Initial condition	Boundary condition
***Deformation***	
*w* (*x*, *y*, *z*, 0) = 0 (0 < *x* < *L*_1_, 0 < *y* < *L*_1_, 0 < *z* < *H*)	*w* (*x*, *y*, *H*, *t*) = 0 (*t* ≥ 0) *w* (0, *y*, *z*, *t*) = *w* (*L*_1_, *y*, *z*, *t*) = 0 (*t* ≥ 0) *w* (*x*, 0, *z*, *t*) = *w* (*x*, *L*_1_, *z*, *t*) = 0 (*t* ≥ 0)
***Pore fluid flow***	
*u* (*x*, *y*, *z*, 0) = 50 (0 < *x* < *L*_1_, 0 < *y* < *L*_1_, 0 < *z* < *H*)	*u* (*x*, *y*, 0, *t*) = *u* (*x*, *y*, *H*, *t*) = 0 ( *t* ≥ 0) *u* (0, *y*, *z*, *t*) = *u* (*L*_1_, *y*, *z*, *t*) = ∂*u*/∂*x* = 0 (*t* ≥ 0) *u* (*x*, 0, *z*, *t*) = *u* (*x*, *L*_1_, *z*, *t*) = ∂*u*/∂*y* (*t* ≥ 0)
***Solute transport***	
*c* (*x*, *y*, *z*, 0) = 0 kg/m^3^ (0 < *x* < *L*_1_, 0 < *y* < *L*_1_, 0 < *z* < *H*)	*c* (*x*, *y*, 0, *t*) = *c*_0_,* c* (*x*, *y*, *H*) = 0 (*t* ≥ 0) *c* (0, *y*, *z*, *t*) = *c* (*L*_1_, *y*, *z*, *t*) = ∂*c*/∂*x* (*t* ≥ 0) *c* (*x*, 0, *z*, *t*) = *c* (*x*, *L*_1_, *z*, *t*) = ∂*c*/∂*y* (*t* ≥ 0) *c*_0_ = 58.5 kg/m^3^

**Fig. 3:**
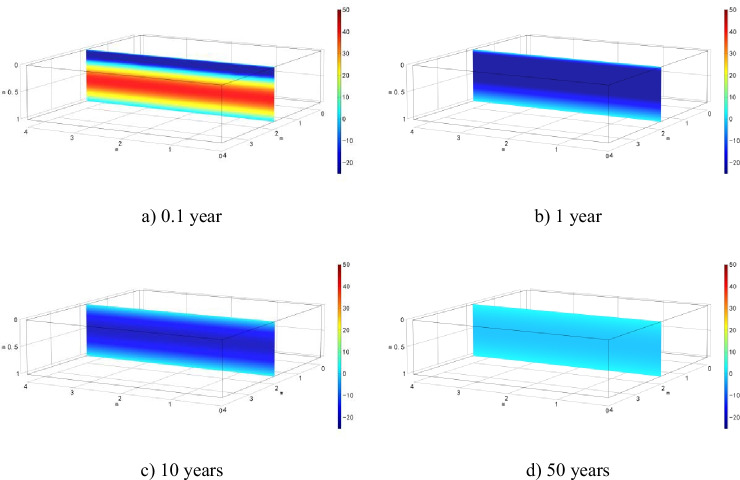
Evolution of excess pore water pressure in semi-permeable clay liner after

Evolutions of excess pore fluid pressure in the Case 1 model domain are shown in Fig. 3. The cloud diagrams of *u* correspond to the results after 0.1, 1.0, 10, 20, and 50 years of simulation.

**Fig. 4:**
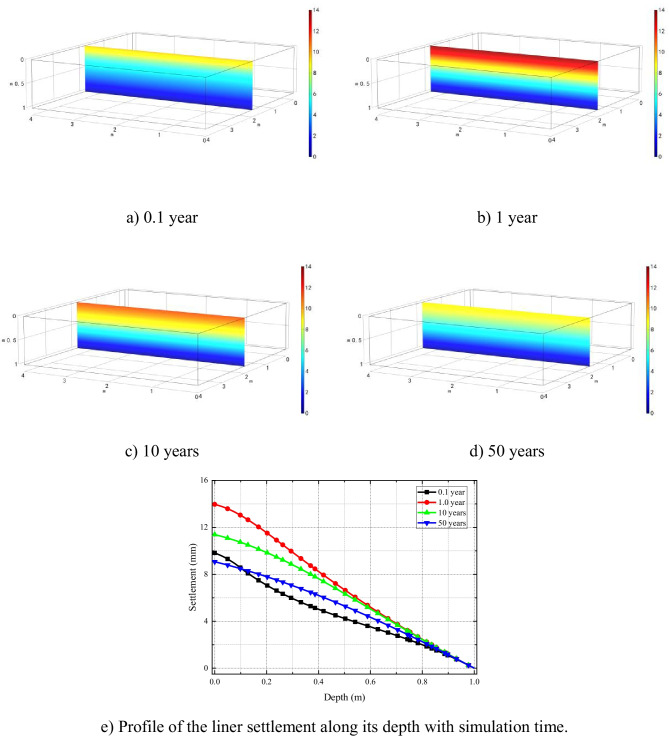
Evolution of the clay liner settlement after

The cloud diagrams of clay liner settlement at different times are shown in Fig. 4(a)–(d). The settlement profiles along the length of the liner depth with time are presented in Fig. 4(e). Two stages, e.g., (I) the increasing period and (II) the decreasing period, can be clearly seen from Fig. 4. The reason why the settlement evolution presents two stages is because mechanical consolidation and chemico-osmosis consolidation are key factors controlling soil deformation which occur successively with simulation time. As mentioned previously that, at the initial stages, mechanical consolidation is the dominant factor affecting soil settlement, and with the dissipation of positive excess pore water pressure, soil deformation continues. However, with time, mechanical consolidation is completed, and chemico-osmosis consolidation caused by chemical loading dominates. Meanwhile, as the solute transport continues into the clay liner, solute concentration difference across the semi-permeable clay gradually decreases, which reduces the degree of chemico-osmosis consolidation, leading to the rebound of soil deformation predicted in stage (II).

**Fig. 5:**
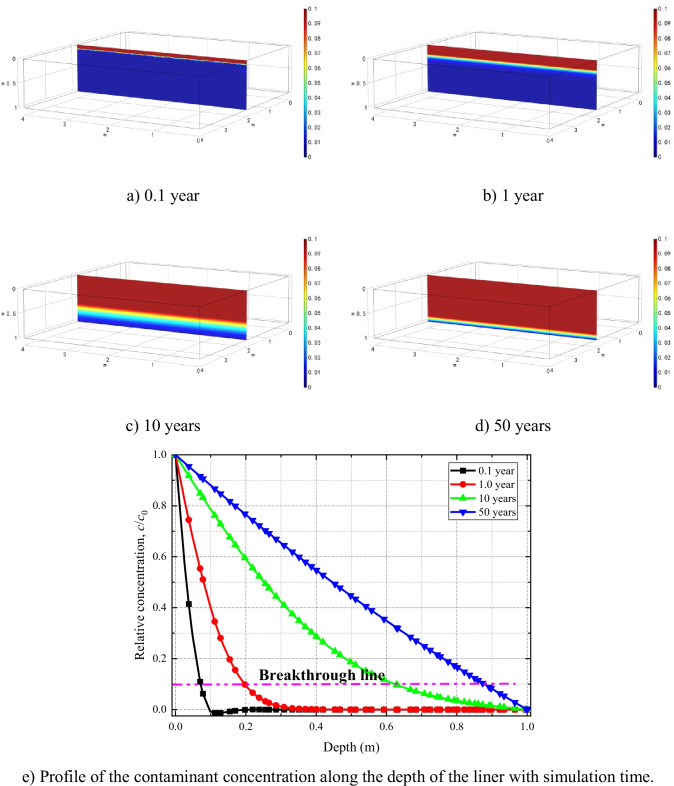
Evolution of solute concentration distributions after

The cloud diagrams of solute concentration distribution at 0.1, 1.0, 10, 20, and 50 years are shown in Fig. 5. The results predict that the solute transport continues downward with time and the penetration distance of the solute at 10 years is 0.62 m, and at 50 years, the solute penetrates the entire clay liner. The penetration distances of solute are one of the most important parameters for estimating the liner performance, which is defined as the distance where the in situ solute concentration reaches 10% of the source concentration, i.e., *c*/*c*_0_ = 0.1 (Fig. 5e). Solute concentration at the half-length of the liner depth reaches to 45% of that of the source after 50-year simulation period.

### Barrier system including geomembrane-compacted clay composite liner (Case 2)

In this case, the clay liner is exposed to leachate via a damaged GMB/CCL, which is illustrated in Fig. 1b. The initial and boundary conditions of the simulations are presented in Table 5.

**Table 5:** Initial and boundary conditions of the Case 2 (GMB/CCL) simulation scenario

Initial condition	Boundary condition
***Deformation***	
*w* (*x*, *y*, *z*, 0) = 0 (0 < *x* < *L*_1_, 0 < *y* < *L*_1_, 0 < *z* < *H*)	*w* (*x*, *y*, *H*, *t*) = 0 (*t* ≥ 0) *w* (0, *y*, *z*, *t*) = *w* (*L*_1_, *y*, *z*, *t*) = 0 (*t* ≥ 0) *w* (*x*, 0, *z*, *t*) = *w* (*x*, *L*_1_, *z*, *t*) = 0 (*t* ≥ 0)
***Pore fluid flow***	
*u* (*x*, *y*, *z*, 0) = 50 (0 < *x* < *L*_1_, 0 < *y* < *L*_1_, 0 < *z* < *H*)	*u* (*x*, *y*, 0, *t*) = *u* (*x*, *y*, *H*, *t*) = 0 (*t* ≥ 0) *u* (0, *y*, *z*, *t*) = *u* (*L*_1_, *y*, *z*, *t*) = ∂*u*/∂*x* = 0 (*t* ≥ 0) *u* (*x*, 0, *z*, *t*) = *u* (*x*, *L*_1_, *z*, *t*) = ∂*u*/∂*y* (*t* ≥ 0)
***Solute transport***	
*c* (*x*, *y*, *z*, 0) = 0 (0 < *x* < *L*_1_, 0 < *y* < *L*_1_, 0 < *z* < *H*)	*c* (*x*, *y*, 0, *t*) = *c*_0_ ((*x*, *y*, 0) $\in$ Ω_2_), *c* (*x*, *y*, 0, *t*) = 0 ((*x*, *y*, 0) $\notin$ Ω_2_), *c* (*x*, *y*, *H*) = 0 (*t* ≥ 0) *c* (0, *y*, *z*, *t*) = *c* (*L*_1_, *y*, *z*, *t*) = ∂*c*/∂*x* (*t* ≥ 0) *c* (*x*, 0, *z*, *t*) = *c* (*x*, *L*_1_, *z*, *t*) = ∂*c*/∂*y* (*t* ≥ 0) *c*_0_ = 58.5 kg/m^3^

**Fig. 6:**
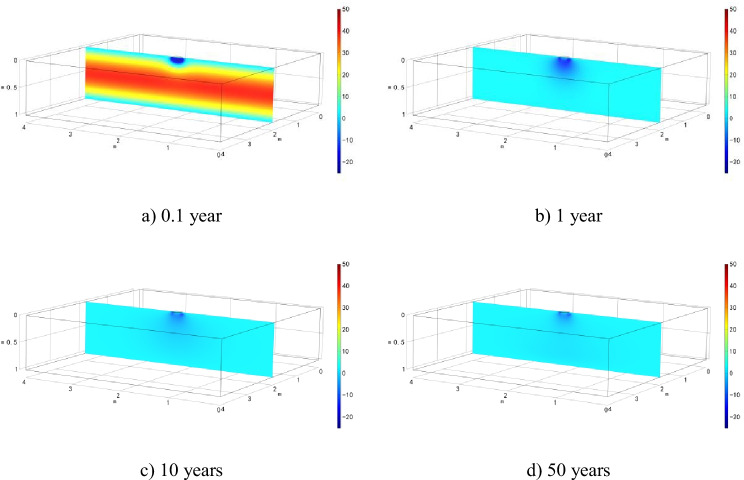
Evolution of excess pore water pressure in semi-permeable clay liner after

The cloud diagrams of excess pore fluid pressure evolution under local leakage condition are presented in Fig. 6. The results show that during the early simulation period (0.1 year), excess pore fluid pressure is positive everywhere except around the leakage area. The positive pore fluid pressure is associated with the mechanical consolidation. However, the excess positive pressure disappears rapidly, and negative pressure develops due to chemical-osmosis.

**Fig. 7:**
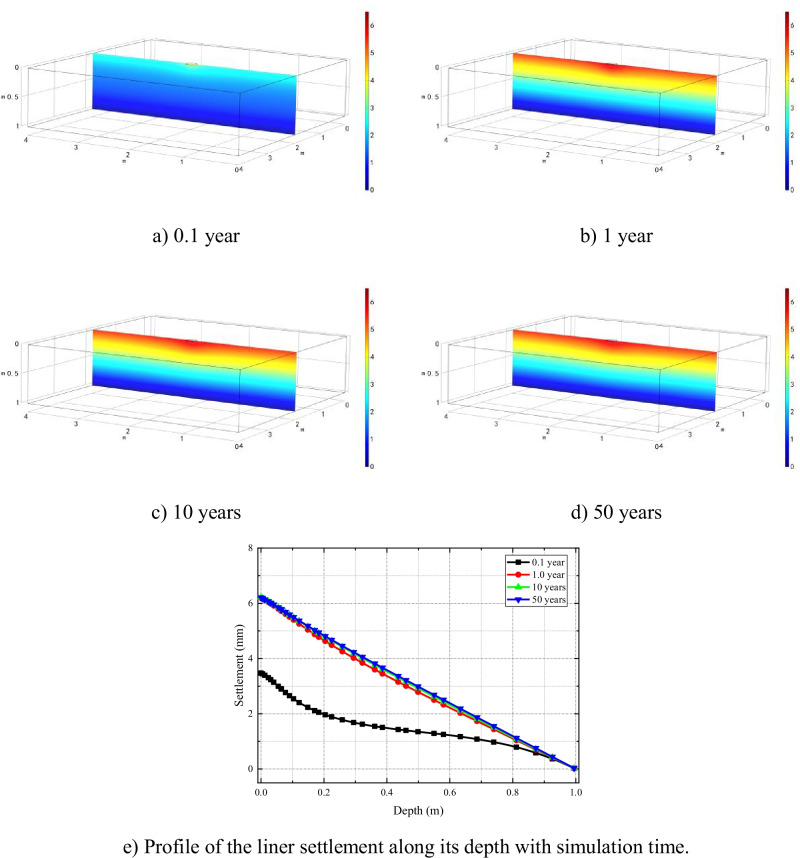
Evolution of excess pore water pressure in semi-permeable clay liner after

Figure 7 shows the settlement along the depth of the clay liner at different simulation time under local leakage condition. The settlement of clay liner directly below the leakage area, which is controlled by mechanical consolidation and chemical-osmotic consolidation, is greater than that of surrounding soil. Furthermore, with the completion of consolidation, the soil settlement increases gradually, reaches the maximum in about 1 year, and seems to change marginally afterwards.

**Fig. 8:**
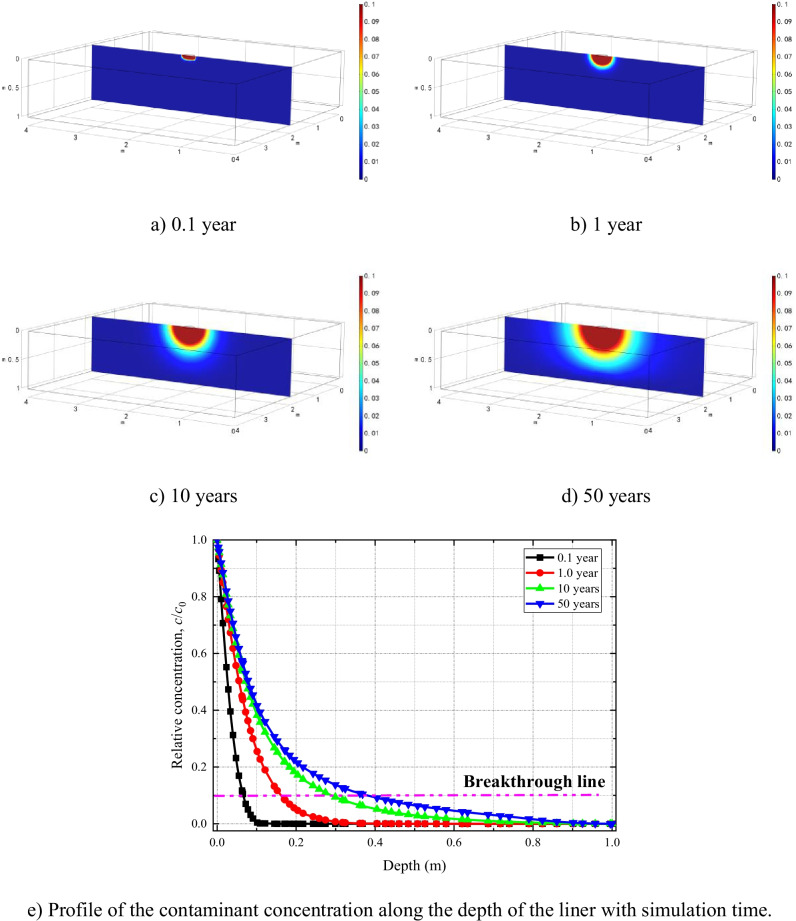
Evolution of solute concentration distributions after

Figure 8 shows the spread of the solute in the model domain for various Case 2 simulation periods. After 50 years, horizontal spread of the solute, at the upper surface of the clay liner, reaches to 0.29 m, and vertical penetration to around 0.382 m. The results show significant improvement of liner performance due to consideration of the geomembrane. The breakthrough concentration (*c*/*c*_0_ = 0.1) reaches only up to 0.4 m of the depth of the liner for the duration of the simulation period that is significantly less than the predicted concentration in Fig. 5(e). Solute breakthrough, across the depth of the clay liner, did not occur during the 50-year simulation period.

### Influence of geomembrane on hydraulic-mechanical-chemical response of clay liner

In order to investigate the influence of geomembrane on hydraulic-mechanical-chemical response of clay liner, evolution of excess pore water pressure, soil deformation, and solute transport at the center line of clay liner for the Case 1: compacted clay liner system and Case 2: geomembrane-compacted clay composite liner system, have been compared here.

**Fig. 9:**
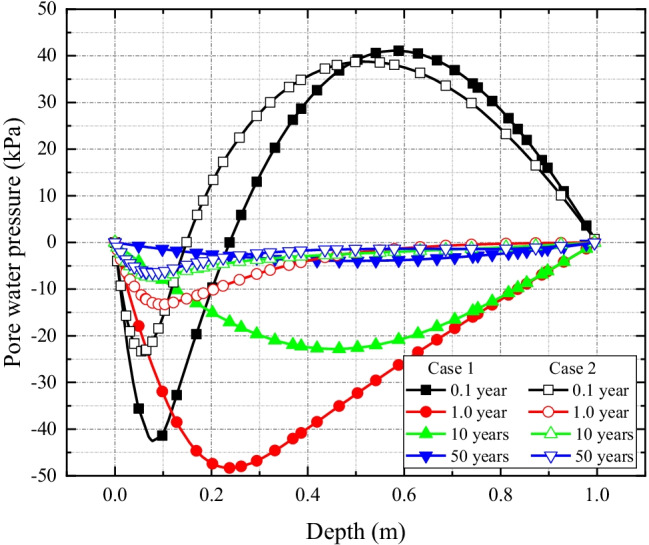
Comparison of excess pore water pressure profiles along the depth of the liner for Case 1 and Case 2 simulation scenarios

The comparison of excess pore fluid pressure for the two cases are presented in Fig. 9. It shows that the negative pressure corresponding to Case 2 is less than that of Case 1. This is because the chemical loading at the upper boundary of clay liner in Case 2 is much smaller than that in Case 1. Furthermore, it is worth noting that the dissipation rate of excess pore pressure in Case 2 is slower, and the peak negative pressure occurs at depth around 0.1 m during the simulation period while, in Case 1, it moves progressively from 0.1 to 0.45 m along the depth of the liner. This variation reflects the importance of multi-dimensional hydrodynamic dispersion in solute transport, which occurs in both vertical and horizontal directions in Case 2 and weakens the spread or reduction of the concentration difference inside and outside the clay liner. This eventually slows down dissipation of negative excess pore pressure.

**Fig. 10:**
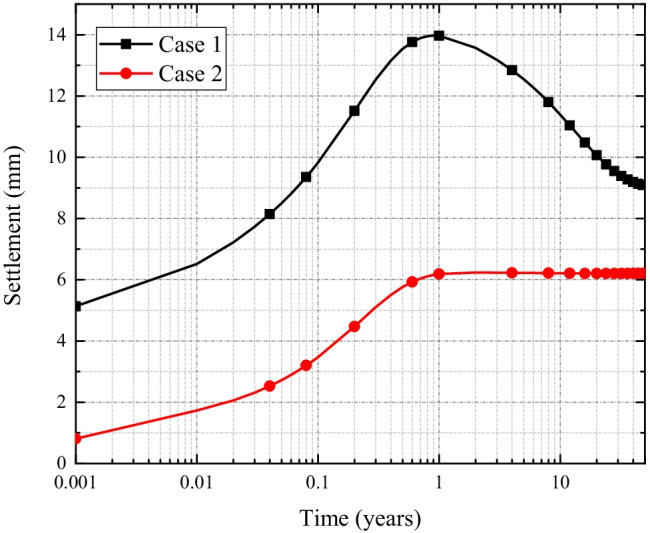
Comparison of liner settlement profiles along the depth of the liner for Case 1 and Case 2 simulation scenarios

The results of time-dependent settlement behavior of Case 1 and Case 2 simulation scenarios are presented in Fig. 10. The graphs of Case 1 exhibit an initial (I) increasing phase, followed by a (II) decreasing phase which is, however, not visible in Case 2. The settlement in Case 1 is larger than that of the Case 2, since the greater chemical loading at the upper boundary of the liner in Case 1 causes stronger chemico-osmotic consolidation than that of the Case 2. Furthermore, compared to Case 2, the solute transport rate in Case 1 is faster, which greatly reduces the chemical loading, resulting in the rebound of soil settlement observed in the results. The results demonstrate the usage of geomembrane on reducing the soil settlement, rebound, and undulation.

**Fig. 11:**
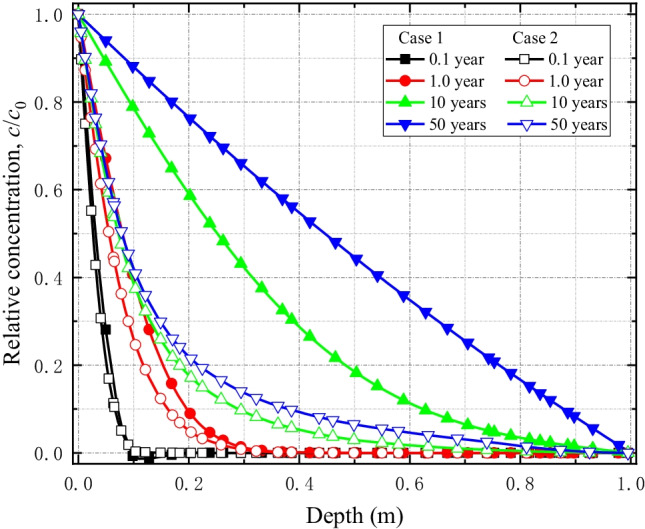
Comparison of solute concentration profiles along the depth of the liner for Case 1 and Case 2 simulation scenarios

Figure 11 shows the graphs of solute concentration with depth in Cases 1 and 2. Expectedly, the solute transport is faster in Case 1 than Case 2. The reason is that in Case 1, contaminant loading is uniform at the *xy*-plane which results into higher contaminant flux than Case 2 where the load is applied as a point load. Also, in Case 2, both longitudinal and transverse spreads result into slower movement of the contaminant which experiences only one directional (longitudinal) spread, i.e., along the *z*-axis in Case 1 due to uniform loading condition.

### Significance of model dimensionality on predicted results

To evaluate the influence of dimensionality on the coupled HMC response of the clay liner, excess pore water pressure, solute transport, and/or soil deformation results under one-dimensional and three-dimensional conditions are compared.

#### Comparison of 1D vs 3D coupled HMC model for compacted clay liner system

**Fig. 12:**
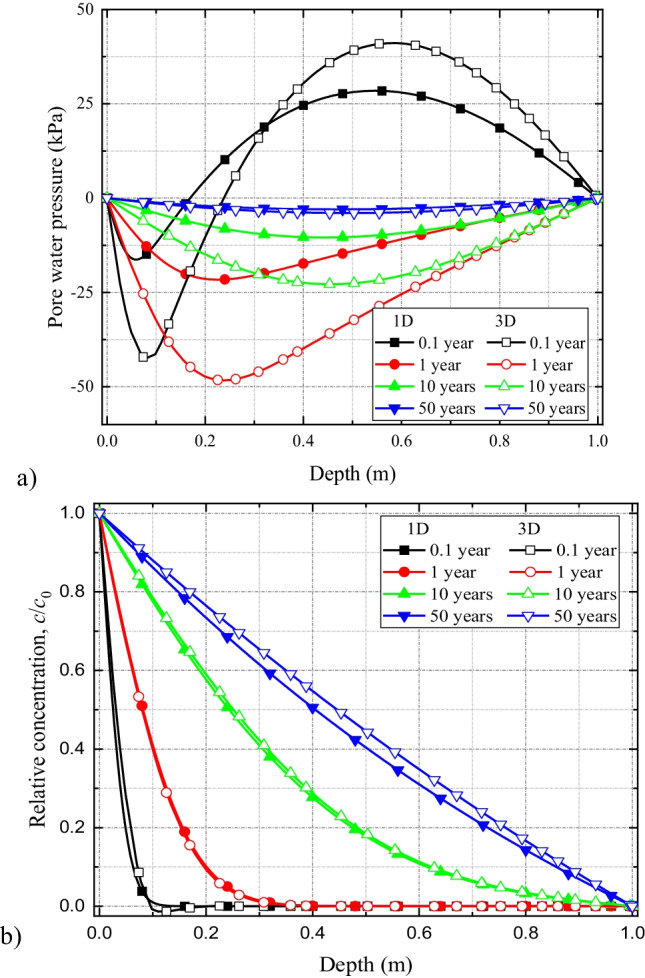
Comparison of (**a**) excess pore water pressure and (**b**) solute concentration profiles along the depth of the liner for 1D and 3D coupled models in single compacted clay liner system

The comparison of excess pore fluid pressures, soil deformation, and solute spread results for the 1D and 3D HMC models are presented in Fig. 12(a)–(c). From the results, it is evident that in compacted clay liners, under uniform contaminant-loading conditions, contaminant spreads almost similarly for both 1D and 3D simulations. Although, at the early stages, higher amount of pore fluid dissipation (or negative pressure development) occurs through more available fluid boundaries during 3D simulations; at the later stages, the fluid pressures become analogous (Fig. 12a). The results suggest that for uniform contaminant-loading conditions, the one-dimensional and three-dimensional model behaviors are similar for simple liner systems.

#### Comparison of the simulation results of 1D and 3D HMC coupled models in geomembrane-compacted clay composite liner system

**Fig. 13:**
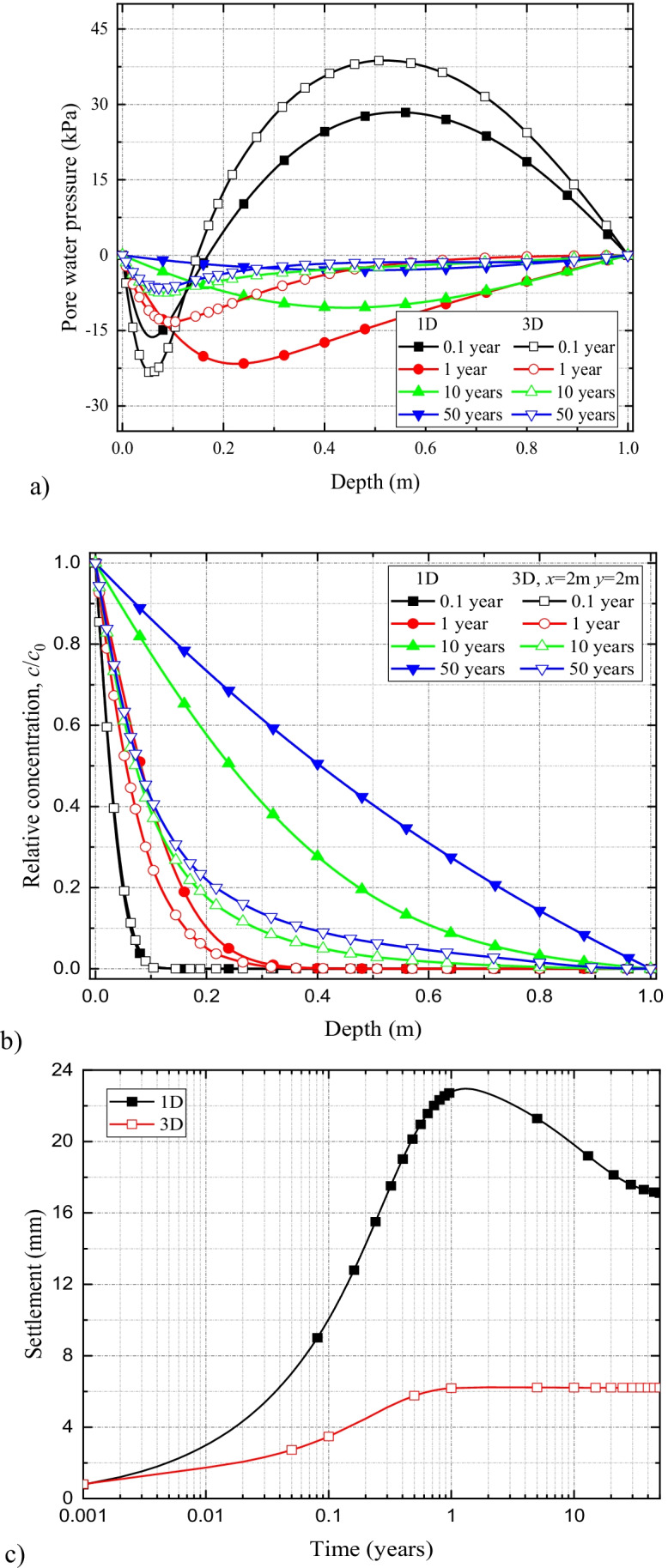
Comparison of (**a**) excess pore water pressure, (**b**) solute concentration, and (**c**) settlement profiles with time for 1D and 3D coupled models in geomembrane-compacted clay composite liner system

The comparison of excess pore fluid pressure developments for 1D and 3D HMC coupled models in the geomembrane-compacted clay composite liner system is presented in Fig. 13a. It shows that, at the initial stages (0.1 year), the negative pressure development in the 3D coupled model is expectedly greater than that of the 1D model due to higher dissipation of pore fluid through the more available fluid boundaries (Fig. 13a). Although with simulation time, the variations between the two pore fluid pressure profiles (1D vs 3D) are reduced, the deviations, especially at the vicinity of the contaminant source, remain largely noticeable for the damaged GMB/CCL system. In comparison, for the CCL system, the deviations between the two profiles became negligible (Fig. 12a).

The predicted solute concentration values corresponding to the 1D HMC coupled model are greater than those of the 3D model (Fig. 13b). The reason is that contaminants migration in the transverse direction is not considered in 1D models which eventually accelerate its spread only in the longitudinal direction (in this case, the vertical *z*-direction). This suggest that the 1D coupled models significantly overestimate solute transport comparing to the 3D models.

Results of the time-dependent settlement behavior of the GMB/CCL liner are presented in Fig. 13(c). It shows that the peak settlement of the liner, in the 3D HMC coupled model, is 6 mm which is roughly 26% of the peak settlement (23 mm) observed during the 1D simulations. That means, analogous to the contaminant spread, liner settlement is also overestimated during 1D simulations for damaged GMB/CCL systems.

## Conclusions

In this study, a fully coupled 3D hydro-mechanical-chemical (HMC) model is presented to investigate long-term solute transport, soil settlement, and excess pore water pressure evolution in landfill clay liners. The multi-physics finite element software COMSOL Multiphysics was used to develop the model and conduct model simulations. The application of the model focused on solute transport and liner deformation behaviors in a single compacted clay liner and a geomembrane-compacted composite clay liner subjected to leachate leakage conditions.

The results suggest that under field conditions, during osmosis, fluid can flow in all directions across semi-permeable membranes (i.e., clays) and the phenomenon is accurately represented by a 3D model. Lower dimensional models are limited to specific directional flow and therefore lacks true representation of field conditions. This is further reflected on solute spread results predicted in the liner through a damaged geomembrane. Despite the same source concentration, spread of solute at a certain period of time in 3D simulations seems to be less than that of the lower dimensional model simulation. Effect of model dimensionality on solute spread for CCL is found to be marginal, but for GMB/CCL, the effect is significantly large. After 50-year simulation period, solute concentration at the half-length of the GMB/CCL liner is predicted to be 40% of the source concentration during 1D simulation, which is only 6% during the 3D simulation. Solute spread accelerates vertically than horizontally, since overburden load and consequent mechanical loading-induced solute convection occur in the same direction despite the soil being homogeneous and isotropic. Moreover, the results revealed approximately 74% over-estimation of liner settlement in 1D simulation than that of the 3D for GMB/CCL system. This highlights the importance of a three-dimensional hydro-mechanical-chemical model to study solute transport under mechanical and chemico-osmotic loading conditions in semi-permeable clays. The application of the model revealed the utility of geomembrane in compacted clay liners on reducing the soil settlement, rebound, and/undulation as well as restricting the spread of the solute in the liner.

## Data Availability

The data presented in this manuscript are available on request to the first author and the second author.
